# Responsiveness of Various Exercise-Testing Protocols to Therapeutic Interventions in COPD

**DOI:** 10.1155/2013/410748

**Published:** 2013-01-28

**Authors:** Benoit Borel, Steeve Provencher, Didier Saey, François Maltais

**Affiliations:** ^1^Centre de Recherche, Institut Universitaire de Cardiologie et de Pneumologie de Québec, Université Laval, Quebec City, QC, Canada; ^2^Centre de Pneumologie, Institut Universitaire de Cardiologie et de Pneumologie de Québec, 2725 Chemin Sainte-Foy, Quebec City, QC, Canada G1V 4G5

## Abstract

Exercise intolerance is a key element in the pathophysiology and course of Chronic Obstructive Pulmonary Disease (COPD). As such, evaluating exercise tolerance has become an important part of the management of COPD. A wide variety of exercise-testing protocols is currently available, each protocol having its own strengths and weaknesses relative to their discriminative, methodological, and evaluative characteristics. This paper aims to review the responsiveness of several exercise-testing protocols used to evaluate the efficacy of pharmacological and nonpharmacological interventions to improve exercise tolerance in COPD. This will be done taking into account the minimally important difference, an important concept in the interpretation of the findings about responsiveness of exercise testing protocols. Among the currently available exercise-testing protocols (incremental, constant work rate, or self-paced), constant work rate exercise tests (cycle endurance test and endurance shuttle walking test) emerge as the most responsive ones for detecting and quantifying changes in exercise capacity after an intervention in COPD.

## 1. Introduction

Chronic airflow limitation is the defining physiological feature of Chronic Obstructive Pulmonary Disease (COPD) whose main symptom is dyspnea. Exercise intolerance is another major consequence of COPD, leading to a sedentary life style and poor quality of life [1, 2]. In fact, exercise intolerance is central to the progression of the downward spiral of COPD [3]. Considering the key role of exercise intolerance in the pathophysiology and course of COPD, the evaluation of exercise tolerance should now be included in the assessment of this disease [4], especially for the evaluation of the response to pharmacological and nonpharmacological interventions [5–7]. Also, the heterogeneity in the mechanisms of exercise intolerance in COPD highlights the importance of comprehensive exercise testing, assessing all systems potentially involved [8].

Exercise testing is currently included in the follow-up of chronic diseases like COPD. Exercise testing can be used to document the severity of pulmonary disease, the functional impact of altered respiratory function and to better understand the physiopathological mechanisms involved in exercise intolerance; this refers to the discriminative characteristic of the test. Exercise testing can also be used to quantify the impact of an intervention to improve exercise tolerance [9, 10] or dyspnea [11, 12] and in the preoperative and pre rehabilitation assessments of patients, corresponding to the evaluative characteristic of the test. A third characteristic corresponds to the reproducibility of the test. Each exercise testing presents different levels of responsiveness, according to their methodology advantages and disadvantages relative to these characteristics. The main focus of the present paper is to review the responsiveness of various exercise testing protocols that are used to assess the effects of pharmacological (bronchodilation) and nonpharmacological (exercise training, surgery) interventions on exercise capacity in COPD.

We will first discuss the concept of the minimal important difference (MID) since its understanding is crucial to place the magnitude of improvement in exercise tolerance following a given intervention into perspective. Then, the specifics of each testing protocol will be presented including the advantages and disadvantages of each individual protocol regarding their properties. This will be followed by a nonsystematic review of the published results related to the testing protocol under consideration. Finally, the main physiological mechanisms explaining the differences in responsiveness across the exercise protocols will be briefly explained. The exercise protocols will be classified into three categories depending on the workload characteristic (incremental or constant) of the exercise protocols and the self-paced methodology.

## 2. The Concept of the Minimal Important Difference

It is now appreciated that statistical and clinical significances are not synonymous and that the interpretation of clinical trials should be done from a broader perspective taking into account both aspects (statistical and clinical) of the treatment effect. The minimal important difference (MID) is a concept defined as “the smallest difference in score of a domain of interest that patients perceive as beneficial and which would mandate, in the absence of troublesome side effects and excessive cost, a change in the patient's management” [13]. Conceptually, the MID provides guidance to help determining whether a given change in a clinical outcome is associated with meaningful improvement for the patient. What is considered meaningful is typically assessed using questionnaires or perception scales (e.g., the 7-point Likert scale).

Three different methods can be used for the determination of MID values [14]: (i) the distribution (statistical) approach which is an estimate based on the distribution around the mean of the scores of the measure of interest in an untreated population (usually half the standard deviation of the changes in the variable of interest [15]); (ii) the anchor (external measure) approach which is an estimate based on the comparison of scores of the measure of interest to other outcome measures (the anchors) for which a MID has been previously determined; and (iii) the opinion approach which gathers opinions of experts, patients, and health-care practitioners about what should be considered as a meaningful change.

Each method could lead to various values and there is no single best methodology for the determination of a valid MID estimate. Therefore, it is recommended to combine them since one can gain confidence in the MID estimation if different methodologies provide similar estimates. Strictly speaking, the current methodologies available to assess the MID provide an estimation of the perception threshold and under most circumstances, it remains to be determined if this perception threshold is important for the patients. One important limitation of the MID concept is that MID values are not relevant for the interpretation of individual results. In fact, MID values are often considered as a “cut-off” threshold for a dichotomous categorization of individual results (“responder” or “non-responder” individuals) after an intervention. However, a misinterpretation could appear by considering any individual showing an improvement after intervention greater than MID as a “responder”, whereas the natural individual variability could already exceed the MID value in the absence of an intervention [16]. This phenomenon highlights the limitation of applying MID values obtained by group data to the interpretation of individual results [16, 17] and therefore requires other tools for the interpretation of individual responses to intervention. Significant effort have been put forward to clarify what should be considered as a meaningful difference in power, duration and distance achieved with therapy during several exercise testing procedures over the past 15 years. Collectively, this work is of great assistance in the interpretation of the changes in exercise tolerance seen with interventions. The respective MID values for each different exercise testing protocol presented in this paper are provided in Table 1.

## 3. Incremental Exercise Tests

Based on the progressive increase of the exercise intensity in a short time duration, incremental exercise testing protocols (also called CardioPulmonary Exercise Testing when coupled with physiological measurements; CPET) are currently considered as the “Gold Standard” method for the evaluation of the degree of exercise limitation and to investigate the mechanisms of exercise limitation. These protocols can be performed on a cycle or a treadmill. They are often used to quantify the changes in exercise tolerance after an intervention [4]. Incremental exercise tests are relatively accessible for the evaluation of COPD patients but, when coupled to physiological measurements, they require expensive equipment, which needs regular maintenance and calibration and qualified staff to overview the tests [9].

### 3.1. Incremental Maximal Cycling Exercise Test

Incremental maximal cycling exercise is modestly responsive to rehabilitation, reflecting that the major benefit of exercise training is not to improve peak exercise capacity [18]. Lacasse et al. [19] reported a mean pooled effect of 8.4 Watts (95% CI: 3.4 to 13.4) in peak exercise work rate across 18 studies of pulmonary rehabilitation, an improvement that is in the range of the MID for this parameter. Reviewing the impact of exercise training on peak exercise capacity, Butcher and Jones [20] reported a 7% to 35% improvement of baseline peak exercise capacity. More recently, in a systematic review designed to compare interval and continuous training, Beauchamp et al. [21] showed an increase in peak exercise capacity of 11 Watts (95% CI: 9 to 13) and 10 (95% CI: 8 to 11) for interval and continuous training respectively. Although the available data suggest that CPET is responsive to demonstrate improvements following pulmonary rehabilitation, this exercise protocol is not the most sensitive tool to evaluate the impact of exercise training (see below).

Bronchodilation, a first-line therapeutic option for the management of the COPD, may improve exercise capacity by reducing expiratory flow limitation and air trapping [22]. The effect of short-acting bronchodilators on peak exercise capacity was evaluated in several studies [23–27]. In some of these studies, the improvement in peak exercise work rate was statistically significant but of uncertain clinical significance as the average improvement ranged from 3 to 7 watts [23, 26, 27] while in others, no significant effect of short-acting bronchodilators on peak exercise capacity were demonstrated [6, 25]. The impact of long-acting beta-2-agonist on peak exercise capacity is in the same order of magnitude [24, 25].

To summarize, incremental exercise testing protocols show a consistent responsiveness to interventions such as rehabilitation or bronchodilation but the magnitude of improvement is small. The modest responsiveness of the incremental exercise testing protocol can be easily understood when considering that peak exercise performance does not dramatically change with interventions, even in the healthy population, and that the spectrum of changes is much greater for endurance exercise capacities than for peak exercise capacities [18]. Furthermore, the absence of a well defined MID value could be a negative point for the responsive characteristic of this type of protocol. Despite this limitation for the assessment of response to therapy, incremental exercise testing is clinically useful in the exploration of the mechanisms of exercise limitation and as discriminative tools in the assessment of patients with COPD.

### 3.2. Incremental Maximal Exercise Test on Treadmill

Incremental maximal exercise tests can also be performed on treadmill. The rationale for doing so is to better mimic daily activities of COPD patients, even if the pattern of walking is different on treadmill than in daily conditions [28]. Only few studies used this methodology to assess patients with COPD [29, 30], and to the best of our knowledge, none used incremental treadmill exercise for assessing the impact of an intervention (pulmonary rehabilitation or bronchodilation).

### 3.3. Incremental Shuttle Walking Test

The ISWT is responsive to exercise training. Griffiths et al. [31] reported an increase in ISWT walking distance, ranging from 140 metres to 211 metres following a 6-week rehabilitation program for the rehabilitation group, a value that is clearly above the MID for this variable. Singh et al. [32] also reported significant improvement in the distance walked during the ISWT but to a lesser extent (+58 metres after a 7-week training program). However, the responsiveness of the ISWT to pulmonary rehabilitation is not universal. One randomized controlled trial aiming to determine the effect of creatine supplementation as an adjunct therapy to exercise training on functional exercise capacity in patients with COPD reported only a modest improvement in ISWT distance of 36.8 metres (95% CI: 17.6 to 56.1) for “Creatine and exercise training” group and of 24.3 metres (95% CI: 7.7 to 40.9) for “exercise training alone” group [33]. This improvement of ISWT distance is consistent in magnitude with the findings of Revill et al. [34], who reported a 37-metre improvement in the distance walked during the ISWT after a 7-week pulmonary rehabilitation.

The incremental shuttle walking test has been also used to evaluate the effects of bronchodilation on exercise capacity in COPD. In general, bronchodilation alone has only a modest impact on ISWT performance, with studies reporting smaller gains in walking distance than the proposed MID value for this exercise testing protocol. Twelve weeks of treatment with formoterol (a long-acting *β*_2_-agonist) or ipratropium (a short-acting anticholinergic), compared to placebo, did not improve ISWT walking distance in patients with moderate to severe COPD [35, 36]. Other bronchodilation studies reported statistically significant results but the clinical significance of the findings are questionable. For example, a single dose of procaterol (a short-acting *β*_2_-agonist) was associated with a modest 37-meter improvement in ISWT walking distance [37]. The administration of salmeterol (a long-acting *β*_2_-agonist) during 52 weeks improved the ISWT walking distance by 30 metres (95% CI: 0 to 60) compared to placebo [38]. The use of once-daily inhaled tiotropium (a long-acting anticholinergic) in a 12-weeks multicentre randomized trial also induced a statistically significant increase of ISWT distance by 33 ± 12 metres (10.8%) on day 42 and by 36 ± 14 metres (11.8%) on day 84 compared with placebo [39].

To summarize, incremental walking shuttle test only presents a modest responsiveness to interventions, with the exception of exercise training for which some studies are positive [31, 32]. The improvement in ISWT walking distance reported in bronchodilation studies is usually within the MID value for this variable.

Based on the findings that we have reviewed, incremental exercise protocols (incremental cycle or treadmill exercise tests and ISWT) are not the ideal methodology to assess response to interventions because of questionable responsiveness. These tests are more appropriate in the evaluation of peak exercise capacity and/or the prescription of training programs. Incremental cycling exercise tests, when performed with cardiopulmonary monitoring, can also be used to investigate the physiological response to exercise. Although similar measurements could also be obtained during the ISWT with portable exercise system, this may be more challenging in walking subjects (see Table 1).

## 4. Constant Work Rate Exercise Protocols

The use of constant work rate exercise tests to quantify the effects of an intervention is increasingly popular due to several advantages of this methodology. Constant work rate endurance protocols are based on externally imposed and constant cycling or walking cadence that the patient has to maintain until exhaustion. The primary endpoint of these protocols is thus the endurance time (or the distance which is a product of the speed and time). These tests are usually performed at a high fraction of peak exercise capacity typically representing 75–85% of peak cycling work rate or 80% of the estimated peak VO_2_ during the incremental shuttle walking test. An implication (and a disadvantage of this) is that the constant work rate protocols have to be performed with the knowledge of the peak cycling or walking capacity. These tests address the fact that increasing endurance capacity is a more natural outcome of exercise training than increasing walking speed during self-paced walks.

### 4.1. Cycling Endurance Test (CET)

The CET is generally considered to be more responsive for detecting acute and long-term improvement in functional capacity after pulmonary rehabilitation than the 6MWT [45–47]. Cambach et al. [45] investigated the effects of exercise training on exercise capacity in a cross-over study design. They reported an overall increase in cycling endurance time of ~7 min with exercise training in comparison to the control intervention. Porszasz et al. [48] reported a ~176% increase (from 6.6 min before training to 18.2 min after training) in endurance time in CET after endurance training. Laviolette et al. [10] reported, just after a 6–12 week rehabilitation program, a mean increase of 198 seconds of the endurance time measured during a CET performed at 80% peak work capacity. The improvement in constant work rate endurance time with pulmonary rehabilitation was maintained one year after the rehabilitation program, albeit to a smaller magnitude (137 seconds for endurance time in comparison with baseline values).

Cycle endurance tests have also been widely used for the evaluation of bronchodilation on exercise tolerance in COPD [22, 23, 49–57]. On average, the improvements seen with pharmacotherapy are smaller than with pulmonary rehabilitation. In most short-acting bronchodilation studies, the improvement in endurance time during CET is within the proposed MID for this parameter [23, 54, 58] although one study is positive both from a clinical and statistical point of view [59]. The impact of long-acting bronchodilator on endurance time during CET appears to be superior to that of short-acting bronchodilators with several investigators reporting improvements in endurance time following long-acting bronchodilation ranging from 90 to 236 seconds [22, 49–52]. This may have to do with the increased efficacy of long-acting bronchodilators but also with the longer duration of the treatment period. Some investigators did not succeed in showing benefits of long-acting bronchodilators on exercise tolerance during CET. These studies were generally relatively small and may have lacked statistical power [55, 56].

In summary, cycle endurance tests are considered as a responsive tool for evaluating the effectiveness of a pharmacological or nonpharmacological intervention in COPD population. Based on these findings, the CET exercise protocol should be viewed as more responsive than incremental cycle exercise test or the 6 min walking distance tests in the evaluation of response to therapy in COPD.

**Figure 1:**
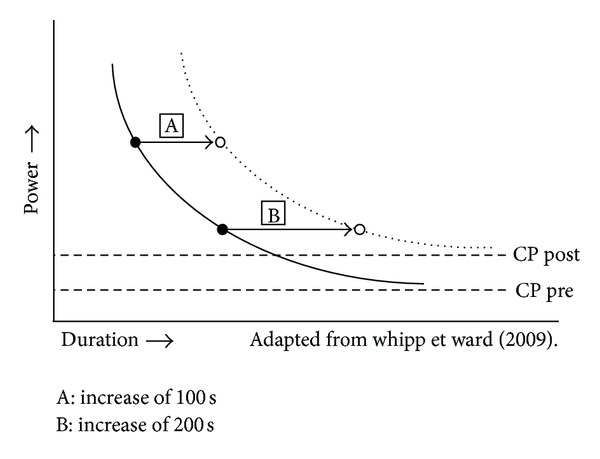
The impact of the power/duration relationship on the exercise endurance response following an intervention. With CP: critical power pre- or postintervention, considered as the asymptote of the power/duration relationship.

One limitation of the CET is that the physiological significance of a given improvement in endurance time after an intervention may be difficult to interpret. This has to do with the power/duration relationship properties that dictate that the endurance time varies exponentially with variations in the workload (or power) that is used during the test [60] and therefore explain the disparity among studies that used singles bouts of endurance test for the evaluation of the effectiveness of an intervention [61]. For example, let us consider the Figure 1 the power/duration relationship before (black circles) and after an intervention (white circles). Assuming that this intervention leads to an increase in critical power corresponding to an upward shift of this parameter (the power or workload for which exercise can be tolerate for very long period of time), the implication is a rightward shift of power/duration relationship, toward higher exercise durations for a given power [61].

From this figure, it can be appreciated that the choice of the power (relative to critical power) at which the test is performed at baseline (CP pre) influences the magnitude of improvement in endurance time seen after the intervention (CP post), and this, for the same degree of physiological improvement [61]. For example, in situation A, performed at a high power output relative to CP pre, the intervention induces an increase of 100 seconds of the CET endurance time. If we consider situation B for which the preintervention endurance time is larger (due to a smaller power output relative to CP pre), the gain in endurance time is now 2-fold higher than with situation A, and this for the same degree of physiological improvement. Therefore, the magnitude of improvement in CET endurance time is dependent on the baseline pretreatment value (i.e., the longer the pretreatment endurance time, the larger the postintervention improvement). Thus, any increase in endurance time to constant load exercise must be interpreted with caution regarding the physiological benefits that have accrued from the intervention, unless the pre- and postintervention power/duration characteristics are also reported [60]. Despite the importance of the power/duration relationship characteristics and its impact on the magnitude of improvement after an intervention, there is no consensus on the intensity to use for the realization of CET. Further investigations are required for the determination of the optimal intensity and therefore offering a standardization which is required for studies comparisons.

### 4.2. Endurance Shuttle Walking Test (ESWT)

The endurance shuttle walk test was developed by Revill et al. and these authors firstly reported the responsiveness of the ESWT to pulmonary rehabilitation [34]. In this study, pulmonary rehabilitation induced a significant improvement amounting to 160% of the ESWT duration after a 7-week pulmonary rehabilitation program (corresponding to an increase of 334 metres). The corresponding improvement in ISWT distance was 32%. Eaton et al. [62] reported a 302-metre increase in ESWT distance after a similar intervention (corresponding to a 92% increase) compared to a 17% improvement (47 m) in the 6MWD. These results highlight the fact that the ESWT may be more responsive to pulmonary rehabilitation than incremental protocols or the 6MWT [62], explaining the growing popularity of the ESWT, and more generally of any constant work rate exercise protocols.

Our group confirmed the responsiveness of the ESWT to detect improvement in walking endurance with various therapies [63, 64]. For example, a 117-second (or 160 metres) increase in endurance time was reported with a single inhalation of salmeterol compared to placebo [63] and a 132-second increase in endurance time was seen after 3 weeks of tiotropium [64]. Thus, similarly to what was reported in pulmonary rehabilitation studies, the ESWT appears to be more responsive than the 6MWT for detecting improvement of exercise tolerance after bronchodilation. This form of exercise is also more relevant to daily activities than cycling exercises [65].

### 4.3. Constant Workrate Treadmill Exercise Test

Constant workrate treadmill exercise test addresses the challenge of the requirement of a corridor to perform the ESWT. This form of exercise has been used in a clinical trial of pharmacotherapy in COPD [66]. The results from this study have been presented in the form of an abstract and full results are in preparation for publication.

### 4.4. 3 Minute Constant Workrate Walking and Stepping Test

A 3 min constant rate shuttle and 3 min constant rate stepping test have recently been developed. These two protocols have been specifically designed to evaluate the effects of therapies on exertional dyspnea, the most prominent symptom in patients with COPD [12, 67]. These tests have the advantage of not requiring an incremental exercise test before their performance since the walking or stepping cadence used during the test is not dependant on peak exercise performance [12, 67]. Their short duration and little requirement for equipment make them suitable for the use in the primary care setting. However, in the study of Perrault et al. [12], multiple bouts of exercise have been performed, limiting the use of such methodology in the primary care setting. Therefore, further studies are required in the future for developing an algorithm which can allow to determine the optimal walking or stepping rate required for inducing a sufficient breathing stimulus and thus reducing the number of exercise bouts. The responsiveness of the 3 min constant rate shuttle walking test to demonstrate reduction in dyspnea following acute bronchodilation has recently been demonstrated is a small clinical trial [67]. Similar results for the 3 min constant rate stepping protocol await confirmation. A MID for these two 3 min exercise protocols has not been determined.

**Figure 2:**
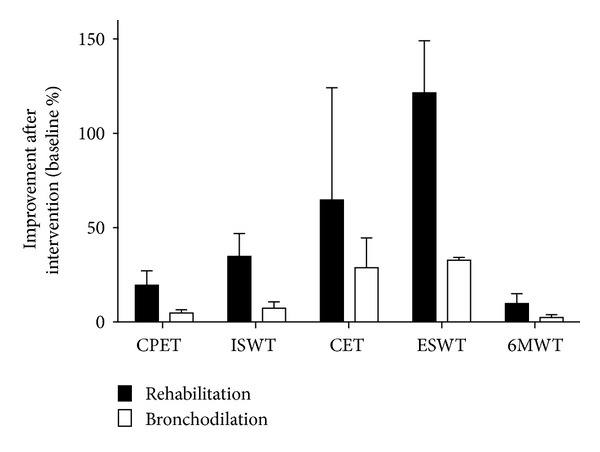
The impact of interventions on the exercise performance following testing methodologies in COPD population. With CPET: CardioPulmonary Exercise Test, ISWT: Incremental Shuttle Walking Test, CET: Cycle Endurance Test, ESWT: Endurance Shuttle Walking Test and 6MWT: 6 minute Walking Test. The percentages of improvement have been calculated, for each exercise protocol, by using the references quoted in the manuscript. All the others tests present in Table 1 (incremental treadmill exercise, constant work rate treadmill test, 3 min walk and step tests, and 6 minute stepper test) are not shown as there is nonexistent or insufficient data in the literature.

The results concerning the responsiveness of the various exercise protocols to interventions are summarized in Figure 2.

In summary, constant work rate exercise protocols appear to be highly responsive to pulmonary rehabilitation and bronchodilation. The magnitude of improvements seen with these protocols after the intervention is well above the MID values, meaning that the observed improvement should be perceived as beneficial by most of the patients. Therefore, this type of testing protocol appears to be an excellent evaluating tool.

## 5. Self-Paced Exercise Tests

Due to the constraints relative to incremental exercise testing protocols and for being more representative of the daily activities performed by COPD patients, field tests have been developed in order to propose more simple tools for the evaluation of exercise capacity. The self-paced walking test, particularly the six-minute walking test, is the most popular field test when it comes to the evaluation of patients with COPD.

### 5.1. Self-Paced Walking Tests

Because of its widespread and ease of application, several pulmonary rehabilitation studies used the 6MWT distance as the main outcome. In a meta-analysis including 11 trials, Lacasse et al. [68] reported a mean treatment effect of 55.7 metres (95% CI: 27.8 to 92.8 metres) between treatment and control groups. In the 2006 updated version of this meta-analysis that involved 16 trials, the common effect for the six-minute walk was 48 metres, with 95% CI: 32 to 65 metres [19]. Similar values have been reported in one study aiming to compare interval and continuous exercise training, with an improvement of 48 metres (95% CI: 29 to 68 metres) after interval training and of 42 metres (95% CI: 25 to 59 metres) after continuous training [21]. Despite its popularity, one study reported that the 6MWT is not the most responsive exercise tool to assess the effects of pulmonary rehabilitation in COPD [10]. This study highlights the fact that 6–12 weeks of pulmonary rehabilitation induce an improvement of the walk distance of 25 ± 52 metres (which is under the MID value). Moreover, the authors report that, among the population under consideration, only 27% of the patients reported an improvement higher than the MID value [10]. In summary, although the 6MWT is responsive to exercise training, it may not be the most responsive test to quantify the exercise-enhancing effects of this intervention.

There is lack of consensus about whether the MID for the 6MWT (and, as a matter of fact, for other exercise protocols) should be expressed in absolute or relative terms. In two studies, the MID value was reported as a percentage of baseline walk distance, ranging from 10% to 14% of the baseline 6 min walk distance [44, 69]. In the field of COPD however, most investigators prefer to report the MID in absolute values assuming that there is a fixed MID value across the range of baseline exercise performance. This practice is also justified based on the fact that an absolute value may be a more sensitive indicator than a relative value [69] and that MID expressed in % baseline value does not necessarily provide a better estimate than the absolute value [7].

The 6MWT has been used to evaluate the effects of bronchodilation in COPD. The overall conclusion is that the 6MWT has a low responsiveness in this setting since most of the studies reported improvement in 6MWD that are well below the MID value for this intervention, ranging from 20 to 42 m with short-acting *β*_2_-agonist [6, 70, 71], from 6 to 39 metres with short-acting anticholinergics [6, 23], from 21 to 54 metres with long-acting *β*_2_-agonist [72–74] and around 10 m with long-acting anticholinergics [75]. The lack of responsiveness of the 6MWT could be due to the presence of a ceiling effect with this test. Although this hypothesis is appealing, it was not supported by the findings of Pepin et al. [76] showing that patients with a high baseline walking distance did not show lesser gains in 6MWD than patients with lower performances. Another hypothesis concerns the intrinsic design and characteristic of the 6MWT, which has a fixed duration and a self-imposed pace. Under this situation, the only way to improve the walking distance is to increase the walking speed, something that patients are not typically incline to do. In fact, patients are likely to tend to repeat the same performance after bronchodilation. This idea is supported by Pepin et al. [76] who reported similar cardiorespiratory kinetics and walking speeds during two separate 6MWT, one being performed after placebo and one after a bronchodilator, confirming that patients tend to reproduce the same walking pattern and to choose similar comfortable walking speed during repeated walking tests, irrespective of the administration of a bronchodilator.

Others interventions have been tested with self-paced walk tests. Inhaled corticosteroids induce an improvement of the walk distance (+33 metres or +8%) [77]. Diaphragmatic strength training [74], lung volume reduction surgery [78] and supplementary oxygen [79] have also been shown to improve walk distance after intervention by 50 to 95 metres (or from 20% to 36%). For these three studies, it is likely that the gains in the 6MWT distance were clinically relevant.

In summary, the current information of the 6MWT indicate that this methodology is not the most responsive to evaluate the effects of interventions (pulmonary rehabilitation or bronchodilation) on exercise tolerance in patients with COPD. However, it is well accepted that self-paced walk tests present a good discriminative capacity for the estimation of the severity of the disease and a good predictive value in estimating vital prognosis [80, 81].

### 5.2. Self-Paced Stepping Test

With the idea of avoiding the need for a corridor and to reproduce stair climbing, an important daily physical task, some authors have developed a 6 min stepping version of the walking protocol [82]. This protocol consists of asking patients to step up and down for as much as possible during a fixed duration of 6 minutes [82]. Little information is available for this specific field test because it has only been recently developed. No MID values are currently available for this test. The responsiveness of the 6 minute stepper test has not been evaluated in the COPD population; the sensitivity of the stepper test being inferred from comparisons of the performance during this test in two COPD versus healthy individuals [82]. Rammaert et al. [83] used the 6 min stepper test for the evaluation of home-based pulmonary rehabilitation in idiopathic pulmonary fibrosis and reported a significant improvement of the number of steps performed after 6 minute of exercise, suggesting that the 6 min stepper test is responsive to rehabilitation. However, this finding could be specific to pulmonary fibrosis and the responsiveness in COPD population needs to be confirmed.

## 6. Physiological Mechanisms Underlying the Responsiveness of Exercise Protocols to Interventions

Aerobic training, alone or in conjunction with strengthening exercises, induces structural changes and adaptations in cardiovascular and muscular systems. These adaptations mainly concern the improvement of oxygen delivery and uptake at exercising muscle level, with adaptations reflecting an increase of muscle capillaries and a conversion from fast fibre type (type II) to slow fibre type (type I), which indicates an increased oxidative capacity of the muscle [84, 85]. As result of these adaptations, the muscle metabolism will be modified promoting the use of the aerobic pathway instead of the glycolytic pathway [86, 87]. One implication of these muscle physiological adaptations will be a reduced tendency toward limb muscle fatigue [88]. The preferential use of aerobic metabolic pathway will also have consequences at the central component of exercise limitation by reducing the ventilatory requirements for a given exercise level [86, 89–91]. The reduced ventilation, along with a slower and deeper breathing pattern [89], will be associated with a decrease of dynamic hyperinflation and dyspnea [48, 92, 93].

The mechanisms of improved exercise tolerance with bronchodilation are different than with exercise training. The use of bronchodilators increases airways calibre, improves expiratory flow rates and, as a consequence, decreases hyperinflation at rest and during exercise [22]. Breathing at lower operative lung volumes will allow larger expansion in tidal volume (*V*_*T*_), a major determinant of exercise tolerance in COPD [94]. This ability to expand *V*_*T*_ reflects lesser mechanical ventilatory constraints in relation to the increased resting and exercising inspiratory capacity and inspiratory reserve volume [49, 95]. From the patient perspective, breathing at lower operative volumes, farther from total lung capacity, has a tremendous impact of the perception of dyspnea [95].

Physiological factors could also explain the different levels of responsiveness of the different tests for the detection of the effectiveness of an intervention (see Figure 2). The better sensibility of walking to detect improvement of exercise tolerance after an intervention could be explained by the fact that walking induces less quadriceps fatigue than during cycling [96]. Leg fatigue is a phenomenon that prevents bronchodilation to fully translate into better exercise capacity [96, 97]. On the other hand, exercise desaturation is more common during walking than cycling [98–100]. This could be viewed as a potential disadvantage of walking protocols since severe hypoxemia could diminish the ability of bronchodilation to improve exercise capacity. Although the ESWT and 6MWT both involve walking they do not share similar responsiveness. This difference in evaluative properties between the two walking protocols is in part related to their design, the main point being that the 6MWT is self-paced and the ESWT externally paced. Therefore, the improvement of the exercise performance for the two tests does not involve the same mechanisms. 6MWT requires the patient to increase his walking speed while the ESWT requires the patient to increase endurance time. Considering the fact that patients tend to reproduce the same walking pattern during 6MWT [76], increasing walking speed with intervention appears more difficult to achieve than walking for a longer period of time at a predetermined cadence.

## 7. Conclusion

This paper focuses on the responsiveness of various exercise testing protocols currently available for the evaluation of exercise tolerance in COPD. It emerges that constant-load endurance tests are generally more responsive for detecting improvement on exercise tolerance after an intervention than incremental exercise tests protocols or self-paced walk tests. However, each test has its own discriminative and evaluative properties (see Table 1) as well as their specific methodological features. As such, the choice of the most appropriate exercise test methodology should be determined according to the objective of the measurement.

## Figures and Tables

**Table 1:** Characteristics of exercise testing protocols in COPD.

Characteristics	Incremental exercise tests			Constant work load exercise tests				Self-paced tests	
	Incremental cycle exercise	Incremental treadmill exercise	ISWT	CET	ESWT	Constant work rate treadmill test	3-min walk and step test	6MWT	6MST
Reproducibility	+	?	?	+++	+	?	+	+	+
Discriminative properties	+++	?	?	?	?	?	?	+++	?
Responsiveness	±	?	±	+++	++	?	?	±	?

MID values	+5–10 W [40, 41]	?	+48 m [42]	+100–200 s [10, 43]	+65 s or +95 m [7]	?	?	+25–54 m [17, 40, 44]	?

With ISWT: Incremental Shuttle Walking Test, CET; Cycle Endurance Test, ESWT: Endurance shuttle Walking Test, 6MWT: 6-minute walking test, 6MST: 6-mminute stepper test; +, ++ and +++: positive result, ±: controversial result, ?: no data in the literature; W: watts, m: meters and s: seconds.

## References

[1] Martinez FJ, Foster G, Curtis JL (2006). Predictors of mortality in patients with emphysema and severe airflow obstruction. *American Journal of Respiratory and Critical Care Medicine*.

[2] Oga T, Nishimura K, Tsukino M, Sato S, Hajiro T (2003). Analysis of the factors related to mortality in chronic obstructive pulmonary disease: role of exercise capacity and health status. *American Journal of Respiratory and Critical Care Medicine*.

[3] Reardon J, Casaburi R, Morgan M, Nici L, Rochester C (2005). Pulmonary rehabilitation for COPD. *Respiratory Medicine*.

[4] American Thoracic Society (2003). ATS/ACCP Statement on cardiopulmonary exercise testing. *American Journal of Respiratory and Critical Care Medicine*.

[5] Bernard S, Whittom F, Leblanc P (1999). Aerobic and strength training in patients with chronic obstructive pulmonary disease. *American Journal of Respiratory and Critical Care Medicine*.

[6] Liesker JJW, Wijkstra PJ, Ten Hacken NHT, Koéter GH, Postma DS, Kerstjens HAM (2002). A systematic review of the effects of bronchodilators on exercise capacity in patients with COPD. *Chest*.

[7] Pepin V, Laviolette L, Brouillard C (2011). Significance of changes in endurance shuttle walking performance. *Thorax*.

[8] Pepin V, Saey D, Laviolette L, Maltais F (2007). Exercise capacity in chronic obstructive pulmonary disease: mechanisms of limitation. *Journal of Chronic Obstructive Pulmonary Disease*.

[9] Aguilaniu B (2010). Impact of bronchodilator therapy on exercise tolerance in COPD. *International Journal of COPD*.

[10] Laviolette L, Bourbeau J, Bernard S (2008). Assessing the impact of pulmonary rehabilitation on functional status in COPD. *Thorax*.

[11] American Thoracic Society (1999). Dyspnea. Mechanisms, assessment, and management: a consensus statement. *American Journal of Respiratory and Critical Care Medicine*.

[12] Perrault H, Baril J, Henophy S, Rycroft A, Bourbeau J, Maltais F (2009). Paced-walk and step tests to assess exertional dyspnea in COPD. *Journal of Chronic Obstructive Pulmonary Disease*.

[13] Jaeschke R, Singer J, Guyatt GH (1989). Measurement of health status: ascertaining the minimal clinically important difference. *Controlled Clinical Trials*.

[14] Make B (2007). How can we assess outcomes of clinical trials: The MCID Approach. *Journal of Chronic Obstructive Pulmonary Disease*.

[15] Norman GR, Sloan JA, Wyrwich KW (2003). Interpretation of changes in health-related quality of life the remarkable universality of half a standard deviation. *Medical Care*.

[16] Dolmage TE, Hill K, Evans RA, Goldstein RS (2011). Has my patient responded? Interpreting clinical measurements such as the 6-minute-walk test. *American Journal of Respiratory and Critical Care Medicine*.

[17] Redelmeier DA, Bayoumi AM, Goldstein RS, Guyatt GH (1997). Interpreting small differences in functional status: the six minute walk test in chronic lung disease patients. *American Journal of Respiratory and Critical Care Medicine*.

[18] Astrand PO, Rodahl K, Dahl HA, Stromme SB (2003). *Textbook of Work Physiology : Physiological Bases of Exercise*.

[19] Lacasse Y, Goldstein R, Lasserson TJ, Martin S (2006). Pulmonary rehabilitation for chronic obstructive pulmonary disease. *Cochrane Database of Systematic Reviews*.

[20] Butcher SJ, Jones RL (2006). The impact of exercise training intensity on change in physiological function in patients with chronic obstructive pulmonary disease. *Sports Medicine*.

[21] Beauchamp MK, Nonoyama M, Goldstein RS (2010). Interval versus continuous training in individuals with chronic obstructive pulmonary disease- a systematic review. *Thorax*.

[22] O’Donnell DE, Fluge T, Gerken F (2004). Effects of tiotropium on lung hyperinflation, dyspnoea and exercise tolerance in COPD. *European Respiratory Journal*.

[23] Oga T, Nishimura K, Tsukino M, Hajiro T, Ikeda A, Izumi T (2000). The effects of oxitropium bromide on exercise performance in patients with stable chronic obstructive pulmonary disease: a comparison of three different exercise tests. *American Journal of Respiratory and Critical Care Medicine*.

[24] Liesker JJW, Van De Velde V, Meysman M (2002). Effects of formoterol (Oxis Turbuhaler) and ipratropium on exercise capacity in patients with COPD. *Respiratory Medicine*.

[25] Akkoca Yildiz O, Onen ZP, Demir G, Eris Gulbay B, Saryal S, Karabiyikoglu G (2006). Is there any difference between effects of ipratropium bromide and formoterol on exercise capacity in moderate COPD patients?. *Tuberkuloz ve Toraks*.

[26] Ikeda A, Nishimura K, Koyama H, Sugiura N, Izumi T (1994). Oxitropium bromide improves exercise performance in patients with COPD. *Chest*.

[27] Ikeda A, Nishimura K, Koyama H, Tsukino M, Mishima M, Izumi T (1996). Dose response study of ipratropium bromide aerosol on maximum exercise performance in stable patients with chronic obstructive pulmonary disease. *Thorax*.

[28] Jenkins S (2008). Which is the best exercise test to assess therapeutic intervention in COPD?. *Chronic Respiratory Disease*.

[29] Hsia D, Casaburi R, Pradhan A, Torres E, Porszasz J (2009). Physiological responses to linear treadmill and cycle ergometer exercise in COPD. *European Respiratory Journal*.

[30] Mathur RS, Revill SM, Vara DD, Walton R, Morgan MDL (1995). Comparison of peak oxygen consumption during cycle and treadmill exercise in severe chronic obstructive pulmonary disease. *Thorax*.

[31] Griffiths TL, Burr ML, Campbell IA (2000). Results at 1 year of outpatient multidisciplinary pulmonary rehabilitation: a randomised controlled trial. *Lancet*.

[32] Singh SJ, Smith DL, Hyland ME, Morgan MDL (1998). A short outpatient pulmonary rehabilitation programme: immediate and longer term effects on exercise performance and quality of life. *Respiratory Medicine*.

[33] Deacon SJ, Vincent EE, Greenhaff PL (2008). Randomized controlled trial of dietary creatine as an adjunct therapy to physical training in chronic obstructive pulmonary disease. *American Journal of Respiratory and Critical Care Medicine*.

[34] Revill SM, Morgan MDL, Singh SJ, Williams J, Hardman AE (1999). The endurance shuttle walk: a new field test for the assessment of endurance capacity in chronic obstructive pulmonary disease. *Thorax*.

[35] Aalbers R, Ayres J, Backer V (2002). Formoterol in patients with chronic obstructive pulmonary disease: a randomized, controlled, 3-month trial. *European Respiratory Journal*.

[36] Wadbo M, Löfdahl CG, Larsson K (2002). Effects of formoterol and ipratropium bromide in COPD: a 3-month placebo-controlled study. *European Respiratory Journal*.

[37] Sukisaki T, Senjyu H, Oishi K, Rikitomi N, Ariyoshi K (2008). Single dose of inhaled procaterol has a prolonged effect on exercise performance of patients with COPD. *Physiotherapy Theory and Practice*.

[38] Stockley RA, Chopra N, Rice L (2006). Addition of salmeterol to existing treatment in patients with COPD: a 12 month study. *Thorax*.

[39] Verkindre C, Bart F, Aguilaniu B (2006). The effect of tiotropium on hyperinflation and exercise capacity in chronic obstructive pulmonary disease. *Respiration*.

[40] Puhan MA, Chandra D, Mosenifar Z (2011). The minimal important difference of exercise tests in severe COPD. *European Respiratory Journal*.

[41] Sutherland ER, Make BJ (2005). Maximum exercise as an outcome in COPD: minimal clinically important difference. *Journal of Chronic Obstructive Pulmonary Disease*.

[42] Singh SJ, Jones PW, Evans R, Morgan MDL (2008). Minimum clinically important improvement for the incremental shuttle walking test. *Thorax*.

[43] Casaburi R (2005). Factors determining constant work rate exercise tolerance in COPD and their role in dictating the minimal clinically important difference in response to interventions. *Journal of Chronic Obstructive Pulmonary Disease*.

[44] Puhan MA, Mador MJ, Held U, Goldstein R, Guyatt GH, Schünemann HJ (2008). Interpretation of treatment changes in 6-minute walk distance in patients with COPD. *European Respiratory Journal*.

[45] Cambach C, Chadwick-Straver RVM, Wagenaar RC, Van Keimpema ARJ, Kemper HCG (1997). The effects of a community-based pulmonary rehabilitation programme on exercise tolerance and quality of life: a randomized controlled trial. *European Respiratory Journal*.

[46] Ong KC, Chong WF, Soh C, Earnest A (2004). Comparison of different exercise tests in assessing outcomes of pulmonary rehabilitation. *Respiratory Care*.

[47] O’Donnell DE, McGuire M, Samis L, Webb KA (1998). General exercise training improves ventilatory and peripheral muscle strength and endurance in chronic airflow limitation. *American Journal of Respiratory and Critical Care Medicine*.

[48] Porszasz J, Emtner M, Goto S, Somfay A, Whipp BJ, Casaburi R (2005). Exercise training decreases ventilatory requirements and exercise-induced hyperinflation at submaximal intensities in patients with COPD. *Chest*.

[49] O’Donnell DE, Voduc N, Fitzpatrick M, Webb KA (2004). Effect of salmeterol on the ventilatory response to exercise in chronic obstructive pulmonary disease. *European Respiratory Journal*.

[50] Maltais F, Hamilton A, Marciniuk D (2005). Improvements in symptom-limited exercise performance over 8 h with once-daily tiotropium in patients with COPD. *Chest*.

[51] O’Donnell DE, Sciurba F, Celli B (2006). Effect of fluticasone propionate/salmeterol on lung hyperinflation and exercise endurance in COPD. *Chest*.

[52] Maltais F, Celli B, Casaburi R (2011). Aclidinium bromide improves exercise endurance and lung hyperinflation in patients with moderate to severe COPD. *Respiratory Medicine*.

[53] O’Donnell DE, Casaburi R, Vincken W (2011). Effect of indacaterol on exercise endurance and lung hyperinflation in COPD. *Respiratory Medicine*.

[54] Oga T, Nishimura K, Tsukino M, Sato S, Hajiro T, Mishima M (2003). A comparison of the effects of salbutamol and ipratropium bromide on exercise endurance in patients with COPD. *Chest*.

[55] Neder JA, Fuld JP, Overend T (2007). Effects of formoterol on exercise tolerance in severely disabled patients with COPD. *Respiratory Medicine*.

[56] Travers J, Laveneziana P, Webb KA, Kesten S, O’Donnell DE (2007). Effect of tiotropium bromide on the cardiovascular response to exercise in COPD. *Respiratory Medicine*.

[57] Man WDC, Mustfa N, Nikoletou D (2004). Effect of salmeterol on respiratory muscle activity during exercise in poorly reversible COPD. *Thorax*.

[58] Aliverti A, Rodger K, Dellacà RL (2005). Effect of salbutamol on lung function and chest wall volumes at rest and during exercise in COPD. *Thorax*.

[59] Peters MM, Webb KA, O’Donnell DE (2006). Combined physiological effects of bronchodilators and hyperoxia on exertional dyspnoea in normoxic COPD. *Thorax*.

[60] Whipp BJ, Ward SA (2009). Quantifying intervention-related improvements in exercise tolerance. *European Respiratory Journal*.

[61] Dolmage TE, Evans RA, Hill K, Blouin M, Brooks D, Goldstein RS (2012). The effect of pulmonary rehabilitation on critical walk speed in patients with COPD: a comparison with self-paced walks. *Chest*.

[62] Eaton T, Young P, Nicol K, Kolbe J (2006). The endurance shuttle walking test: a responsive measure in pulmonary rehabilitation for COPD patients. *Chronic Respiratory Disease*.

[63] Brouillard C, Pepin V, Milot J, Lacasse Y, Maltais F (2008). Endurance shuttle walking test: responsiveness to salmeterol in COPD. *European Respiratory Journal*.

[64] Bédard ME, Brouillard C, Pepin V (2012). Tiotropium improves walking endurance in COPD. *European Respiratory Journal*.

[65] Pitta F, Troosters T, Spruit MA, Probst VS, Decramer M, Gosselink R (2005). Characteristics of physical activities in daily life in chronic obstructive pulmonary disease. *American Journal of Respiratory and Critical Care Medicine*.

[66] Cooper CB, Abrazado M, Legg D, Kesten S (2010). Development and implementation of treadmill exercise testing protocols in COPD. *International Journal of Chronic Obstructive Pulmonary Disease*.

[67] Sava F, Perrault H, Brouillard C (2012). Detecting improvements in dyspnea in COPD using a three-minute constant rate shuttle walking protocol. *Journal of Chronic Obstructive Pulmonary Disease*.

[68] Lacasse Y, Wong E, Guyatt GH, King D, Cook DJ, Goldstein RS (1996). Meta-analysis of respiratory rehabilitation in chronic obstructive pulmonary disease. *Lancet*.

[69] Holland AE, Hill CJ, Rasekaba T, Lee A, Naughton MT, McDonald CF (2010). Updating the minimal important difference for six-minute walk distance in patients with chronic obstructive pulmonary disease. *Archives of Physical Medicine and Rehabilitation*.

[70] Satake M, Takahashi H, Sugawara K (2011). Inhibitory effect of procaterol on exercise dynamic lung hyperinflation during the 6-min walk test in stable patients with chronic obstructive pulmonary disease. *Arzneimittel-Forschung/Drug Research*.

[71] Shioya T, Satake M, Sato K (2008). Long-term effect of the *β*2-receptor agonist procaterol on daily life performance and exercise capacity in patients with stable chronic obstructive pulmonary disease: clinical study with special reference to health-related quality of life and activities of daily living. *Arzneimittel-Forschung/Drug Research*.

[72] Cazzola M, Biscione GL, Pasqua F (2008). Use of 6-min and 12-min walking test for assessing the efficacy of formoterol in COPD. *Respiratory Medicine*.

[73] Boyd G, Morice AH, Pounsford JC, Siebert M, Peslis N, Crawford C (1997). An evaluation of salmeterol in the treatment of chronic obstructive pulmonary disease (COPD). *European Respiratory Journal*.

[74] Weiner P, Magadle R, Berar-Yanay N, Davidovich A, Weiner M (2000). The cumulative effect of long-acting bronchodilators, exercise, and inspiratory muscle training on the perception of dyspnea patients with advanced COPD. *Chest*.

[75] Okudan N, Gök M, Gökbel H, Süerdem M (2006). Single dose of tiotropium improves the 6-minute walk distance in chronic obstructive pulmonary disease. *Lung*.

[76] Pepin V, Brodeur J, Lacasse Y (2007). Six-minute walking versus shuttle walking: responsiveness to bronchodilation in chronic obstructive pulmonary disease. *Thorax*.

[77] Paggiaro PL, Dahle R, Bakran I, Frith L, Hollingworth K, Efthimiou J (1998). Multicentre randomised placebo-controlled trial of inhaled fluticasone propionate in patients with chronic obstructive pulmonary disease. *Lancet*.

[78] Criner GJ, Cordova FC, Furukawa S (1999). Prospective randomized trial comparing bilateral lung volume reduction surgery to pulmonary rehabilitation in severe chronic obstructive pulmonary disease. *American Journal of Respiratory and Critical Care Medicine*.

[79] Leach RM, Davidson AC, Chinn S, Twort CHC, Cameron IR, Bateman NT (1992). Portable liquid oxygen and exercise ability in severe respiratory disability. *Thorax*.

[80] American Thoracic Society (2002). ATS statement: guidelines for the six-minute walk test. *American Journal of Respiratory and Critical Care Medicine*.

[81] Troosters T, Vilaro J, Rabinovich R (2002). Physiological responses to the 6-min walk test in patients with chronic obstructive pulmonary disease. *European Respiratory Journal*.

[82] Borel B, Fabre C, Saison S, Bart F, Grosbois JM (2010). An original field evaluation test for chronic obstructive pulmonary disease population: the six-minute stepper test. *Clinical Rehabilitation*.

[83] Rammaert B, Leroy S, Cavestri B, Wallaert B, Grosbois JM (2011). Home-based pulmonary rehabilitation in idiopathic pulmonary fibrosis. *Revue des Maladies Respiratoires*.

[84] Casaburi R (1994). Physiologic responses to training. *Clinics in Chest Medicine*.

[85] Casaburi R, Zuwallack R (2009). Pulmonary rehabilitation for management of chronic obstructive pulmonary disease. *The New England Journal of Medicine*.

[86] Casaburi R, Patessio A, Ioli F, Zanaboni S, Donner CF, Wasserman K (1991). Reductions in exercise lactic acidosis and ventilation as a result of exercise training in patients with obstructive lung disease. *American Review of Respiratory Disease*.

[87] Maltais F, Leblanc P, Simard C (1996). Skeletal muscle adaptation to endurance training in patients with chronic obstructive pulmonary disease. *American Journal of Respiratory and Critical Care Medicine*.

[88] Mador MJ, Kufel TJ, Pineda LA (2001). Effect of pulmonary rehabilitation on quadriceps fatiguability during exercise. *American Journal of Respiratory and Critical Care Medicine*.

[89] Casaburi R, Porszasz J, Burns MR, Carithers ER, Chang RSY, Cooper CB (1997). Physiologic benefits of exercise training in rehabilitation of patients with severe chronic obstructive pulmonary disease. *American Journal of Respiratory and Critical Care Medicine*.

[90] Maltais F, Leblanc P, Jobin J (1997). Intensity of training and physiologic adaptation in patients with chronic obstructive pulmonary disease. *American Journal of Respiratory and Critical Care Medicine*.

[91] Gagnon P, Bussieres JS, Ribeiro F (2012). Influences of spinal anesthesia on exercise tolerance in patients with COPD. *American Journal of Respiratory and Critical Care Medicine*.

[92] Gigliotti F, Coli C, Bianchi R (2003). Exercise training improves exertional dyspnea in patients with COPD: evidence of the role of mechanical factors. *Chest*.

[93] O’Donnell DE, Revill SM, Webb KA (2001). Dynamic hyperinflation and exercise intolerance in chronic obstructive pulmonary disease. *American Journal of Respiratory and Critical Care Medicine*.

[94] O’Donnell DE, Lam M, Webb KA (1999). Spirometric correlates of improvement in exercise performance after anticholinergic therapy in chronic obstructive pulmonary disease. *American Journal of Respiratory and Critical Care Medicine*.

[95] O’Donnell DE, Hamilton AL, Webb KA (2006). Sensory-mechanical relationships during high-intensity, constant-work-rate exercise in COPD. *Journal of Applied Physiology*.

[96] Saey D, Debigaré R, LeBlanc P (2003). Contractile leg fatigue after cycle exercise: a factor limiting exercise in patients with chronic obstructive pulmonary disease. *American Journal of Respiratory and Critical Care Medicine*.

[97] Deschênes D, Pepin V, Saey D, LeBlanc P, Maltais F (2008). Locus of symptom limitation and exercise response to bronchodilation in chronic obstructive pulmonary disease. *Journal of Cardiopulmonary Rehabilitation and Prevention*.

[98] Cockcroft A, Beaumont A, Adams L, Guz A (1985). Arterial oxygen desaturation during treadmill and bicycle exercise in patients with chronic obstructive airways disease. *Clinical Science*.

[99] Palange P, Forte S, Onorati P, Manfredi F, Serra P, Carlone S (2000). Ventilatory and metabolic adaptations to walking and cycling in patients with COPD. *Journal of Applied Physiology*.

[100] Poulain M, Durand F, Palomba B (2003). 6-Minute walk testing is more sensitive than maximal incremental cycle testing for detecting oxygen desaturation in patients with COPD. *Chest*.

